# Changes in body composition and subsequent cardiovascular disease risk among 5-year breast cancer survivors

**DOI:** 10.3389/fcvm.2023.1259292

**Published:** 2023-11-20

**Authors:** Ji Soo Kim, Jihun Song, Seulggie Choi, Sang Min Park

**Affiliations:** ^1^ International Healthcare Center, Seoul National University Bundang Hospital, Seoul National University College of Medicine, Seoul, Republic of Korea; ^2^ Department of Biomedical Sciences, Seoul National University Graduate School, Seoul, Republic of Korea; ^3^ Department of Internal Medicine, Seoul National University Hospital, Seoul, Republic of Korea; ^4^ Department of Family Medicine, Seoul National University Hospital, Seoul, Republic of Korea

**Keywords:** breast cancer, cardiac, stroke, metabolic, body composition

## Abstract

**Introduction:**

Cardiovascular disease (CVD) remains a leading cause of death in breast cancer survivors, a growing population. The aim of this study was to determine whether changes in body composition, commonly observed in breast cancer survivors, is associated with subsequent CVD risk.

**Methods:**

This cohort study used the Korean National Health Insurance Service database. The study population included 73,271 5-year breast cancer survivors aged 40 years or above. To assess changes in body composition and its effect on the risk of CVD, validated prediction equations and multivariate Cox proportional hazards regression were used. Changes in metabolic markers (blood pressure, total cholesterol, and fasting serum glucose) according to changes in body composition were calculated by multiple linear regression.

**Results:**

Having persistently high predicted lean body and appendicular skeletal muscle mass percentages (LBMP and ASMP, respectively) among breast cancer survivors was associated with 32% and 40% lower CVD risks than a persistently low predicted LBMP or ASMP, respectively. Conversely, persistently high predicted body fat mass percentage (BFMP) was associated with a higher CVD risk than persistently low predicted BFMP. Additionally, those with a low to high change in predicted BFMP had a higher risk of CVD than those with persistently low predicted BFMP. Changes in body composition were accompanied by changes in metabolic markers.

**Discussion:**

Maintaining high percentages of lean body and appendicular skeletal muscle mass and preventing an increase in fat mass may be beneficial in preventing CVD in breast cancer survivors.

## Background

The increased risk of cardiovascular disease (CVD) in breast cancer survivors compared to women without a cancer history is related to the cardiotoxic effects of breast cancer treatment and overlapping risk factors of breast cancer and CVD (1–3). After breast cancer diagnosis, the majority gain weight, which continues, especially in premenopausal women, into breast cancer survivorship (4, 5). Even in the absence of weight gain, changes in body composition consisting of gain in adipose tissue without a gain in or with loss of lean tissue have been observed (6). Chemotherapy also causes alternations in skeletal muscle and creates a predisposition to muscle atrophy and weakness (7). As a result, CVD burden associated with post-diagnosis weight gain has been observed to as long as 5 years in Asian patients (8). Of equal importance, excessive body fat in cancer survivors has been shown to affect quality of life and disease-free survival (9).

Prior research among breast cancer survivors have focused on mortality and body mass index (BMI) or waist circumference (10, 11). In terms of the association between change in BMI and the risk of CVD or CVD mortality, there were contradicting results (12). For example, there was no association between a change in BMI and the risk of CVD in short-term breast cancer survivors. The significance of examining body composition over BMI was further shown in a relatively recent study on adiposity and CVD (13). In this study, the increased risk of CVD was also observed in normal-weight breast cancer survivors with greater visceral adiposity. Research on changes in body composition, distinguishing fat mass and muscle mass, and the risk of CVD in young adults has been carried out, however, little is known on changes in body composition and the risk of CVD in breast cancer survivors (14).

Various anthropometric and imaging indices of obesity and its relationship with CVD risk have been summarized (15). In the current study of 40,095 breast cancer survivors without prior CVD, we utilized validated prediction equations with anthropometric data and health habits to examine the association between changes in body composition and the risk of subsequent CVD.

## Methods

### Data source and study population

In Korea, the National Health Insurance Service (NHIS) has recorded almost all information on medical use in the NHIS database, based on an insurance claim. The NHIS database includes patients’ age, gender, disease history, drug prescription history, and surgery history. The NHIS database can be used for research purposes only by approved researchers (16, 17). In our study, we tracked the risk of CVD in breast cancer patients by accessing the NHIS database through approval of the NHIS.

**Figure 1:**
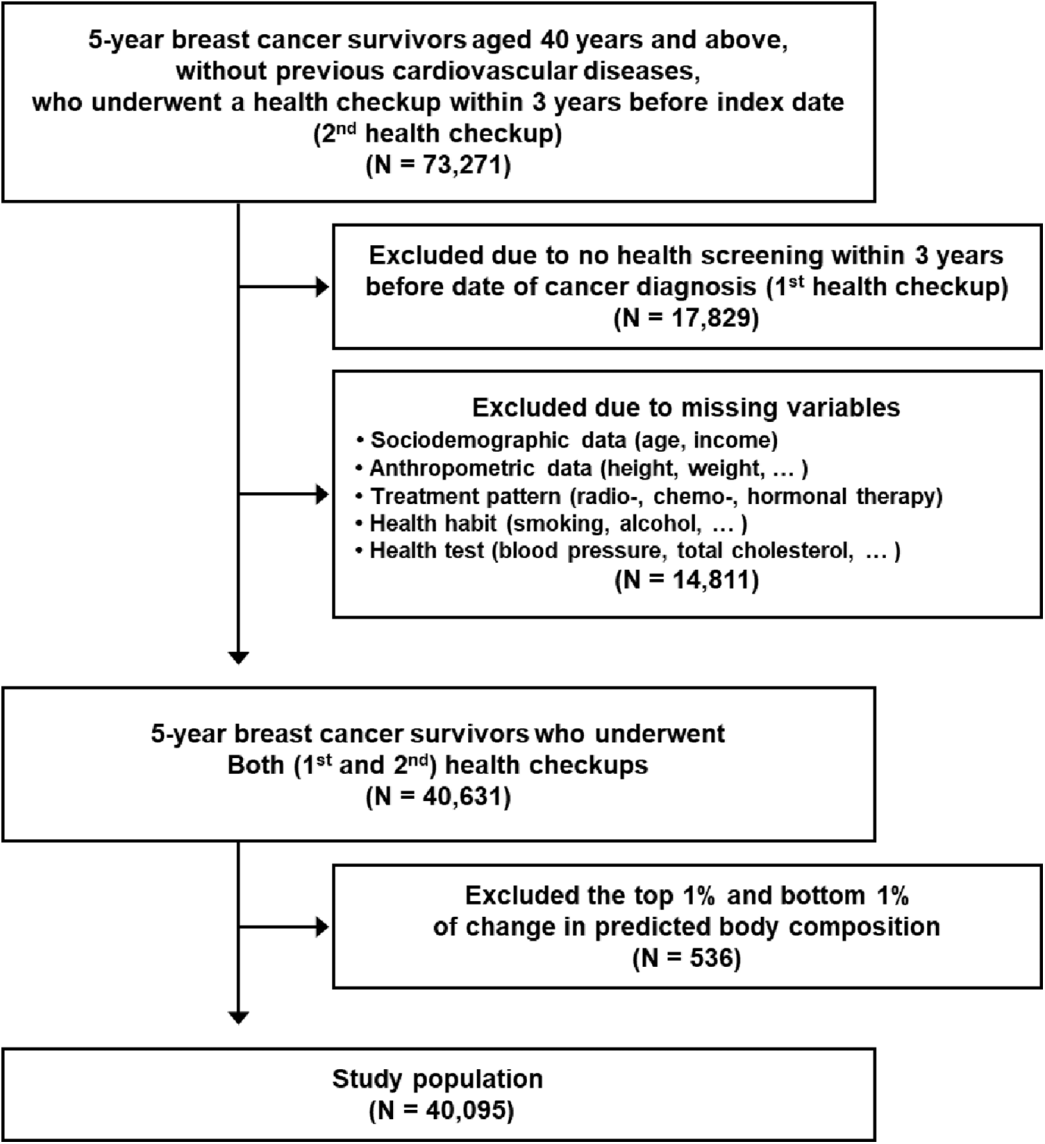
Flow diagram of study population.

73,271 5-year breast cancer survivors aged 40 years or above, without previous CVD, who underwent a health checkup within 3 years before index date (second health checkup) were observed during 2011–2019 (Figure 1). Among them, 17,829 participants were excluded, because they did not take a health screening examination (the first health checkup) during the previous 3 years before the date of initial cancer diagnosis. An additional 14,811 participants who had missing necessary variables to calculate predictive body composition (age, gender, height, weight, waist circumference) and covariates (income level, blood pressure, total cholesterol, etc.) were excluded. Finally, 536 participants with extreme body composition change (top and bottom 1%) were eliminated as cases of outliers (18, 19). As a result, the final study population consisted of 40,095 5-year breast cancer survivors. All participants were followed up from the index date to the date of newly diagnosed CVD, date of death, or 31 December 2020, whichever came first.

### Breast cancer survival and cardiovascular diseases

We recruited all breast cancer patients based on disease information recorded on the NHIS database. Patients diagnosed with breast cancer from 1 January 2006 to 31 December 2014 were extracted based on both the special assessment code (V193 and V194) and the International Classification of Diseases, Tenth Revision (ICD-10; C50) (20). Among 5-year breast cancer survivors, we used ICD-10 codes to identify CVD (I20–I25, I60–I69), coronary heart disease (CHD; I20–I25), and stroke (including ischemic and hemorrhagic stroke; I60–I69) (21, 22). To eliminate events that were not actual CVD events, we defined CVD events as 2 or more days of hospitalization with ICD-10 codes for CVD.

### Key variables

With a validated prediction equation, predicted mass of body composition (kg) and predicted mass index of body composition (kg/m^2^) were assessed and calculated (23). These prediction equations were previously used in studies related to lean body mass, appendicular skeletal muscle mass, and body fat mass (24, 25). Among a total of 40,095 5-year breast cancer survivors, predicted mass index of body composition was evaluated based on age, gender, weight, height, and waist circumference. The percentage of predicted lean body mass (pLBMP), percentage of predicted appendicular skeletal muscle mass (pASMP), and percentage of predicted body fat mass (pBFMP) were then derived at both the first and second health checkups. Changes in body composition (pLBMP, pASMP, and pBFMP) were defined as the difference in the percentage of predicted body composition between the second and first health check-ups; this value shows the difference in body composition before initial cancer diagnosis and after 5 years of survival from breast cancer. At the first health checkup, the High and Low body composition groups were classified based on pre-defined cut-off values: 65% for pLBMP, 26% for ASMP, and 34% for pBFMP. Similarly, the High and Low groups were classified at the second health check-up with the same cut-off value for respective body compositions (26). Finally, according to changes in body composition, a total of 40,095 5-year breast cancer survivors were divided into four groups: those who had consistently low body composition (Low to Low), those who had low body composition before initial cancer diagnosis but high body composition after 5-years of survival (Low to High), those who had high body composition before initial cancer diagnosis but low body composition after 5-years of survival (High to Low), and those who had consistently high body composition (High to High). Additionally, in a sensitivity analysis for changes in body composition, re-classification was performed based on the median of the first (65.01% for pLBMP, 25.74% for pASMP, and 33.85% for pBFMP) and second health checkup (65.13% for pLBMP, 25.69% for pASMP, and 33.73% for pBFMP).

### Statistical analysis

Participants were evaluated for adjusted hazard ratios (aHR) and 95% confidence intervals (95% CI) of the CVD risk according to changes in body composition using multivariate Cox proportional hazards regression after adjustments for the covariates. The considered covariates included age (continuous, years), income level (categorical, first, second, third, and fourth quartiles), smoking status (categorical, never-, past, and current smokers), alcohol consumption (categorical, 0, 1–2, 3–4, and 5 or more times per week), physical activity (categorical, 0, 1–2, 3–4, and 5 or more times per week), BMI (continuous, kg/m^2^), Charlson comorbidity index (CCI; continuous), history of chemotherapy (categorical; cyclophosphamide, trastuzumab, doxorubicin, epirubicin, docetaxel, paclitaxel, and cisplatin), history of radiation therapy, and history of hormone therapy (categorical; tamoxifen, anastrozole, and letrozole). Income level was derived from the insurance premium. BMI was calculated by dividing body weight by the square of height (kg/m^2^). Smoking status, alcohol consumption, and physical activity were assessed by a self-reported questionnaire at the health check-up. The algorithm for calculating Charlson comorbidity index was adapted from a previous study (27). Prescription of anti-cancer drugs known to cause heart disease was collected on the NHIS database (28). Through insurance claims, history of radiation treatment was also collected (29). Chi-squared tests for categorical variables and analysis of variance for continuous variables were used to compare the differences in the distribution of covariates. Blood pressure (mmHg), total cholesterol (mg/dl), and fasting serum glucose (mg/dl) are known to cause CVD (30). Change in blood pressure (systolic blood pressure (sBP) and diastolic blood pressure (dBP)), total cholesterol (TC), and fasting serum glucose (FSG) according to changes in body composition was evaluated with adjusted mean and 95% CI, which was calculated by multiple linear regression after adjustments for the following covariates: age, income, chemotherapy, radiation therapy, hormone therapy, CCI, smoking status, alcohol consumption, and physical activity. A stratified analysis of the association of change in body composition was then performed with the overall CVD events according to age, CCI, and treatment pattern (chemotherapy, hormone therapy, and radiation therapy). Statistical significance was defined as *p*-value < 0.05. *p*-value by Chi-square test for categorical variables and analysis of variance (ANOVA) for continuous variables were used to determine the risk of CVD. All data collection and statistical analyses were conducted using SAS 9.4 (SAS Institute Inc., Cary, NC, USA).

## Results

Table 1 shows the characteristics of the study population, based on change in percentage of predicted lean body mass. Mean ages(standard deviation) for Low to Low, Low to High, High to Low, and High to High groups were 59.99 (9.18), 58.86 (8.71), 56.38 (8.56), and 55.25 (8.10), respectively. The majority of subjects were never-smokers with rare alcohol consumption and physical activity. In addition, the majority of subjects received chemotherapy, radiation therapy, or hormone therapy.

**Table 1:** Descriptive characteristics of the study population.

	Change in percentage of predicted lean body mass				*p*-value
	Low % at baseline periods		High % at baseline periods		
	Low to Low	Low to High	High to Low	High to High	
Study population, *N*	16,462	3,497	2,890	17,246	
% at baseline period, means (SD)	62.14 (1.87)	64.00 (0.84)	65.92 (0.81)	67.84 (2.01)	<0.001
% at follow-up period, means (SD)	62.19 (1.89)	66.05 (0.88)	64.01 (0.84)	67.99 (2.08)	<0.001
Change in %, means (SD)	0.05 (1.30)	2.06 (1.10)	−1.91 (1.05)	0.16 (1.56)	<0.001
Change in %, range	−4.93, 4.95	0.00, 4.98	−4.95, −0.02	−4.99, 5.00	
Age [years], mean (SD)	59.99 (9.18)	58.86 (8.71)	56.38 (8.56)	55.25 (8.10)	<0.001
Age [years], *N* (%)					<0.001
40–49	2,122 (12.9)	495 (14.2)	637 (22.0)	4,511 (26.2)	
50–59	6,405 (38.9)	1,546 (44.2)	1,357 (47.0)	8,252 (47.8)	
≥60	7,935 (48.2)	1,456 (41.6)	896 (31.0)	4,483 (26.0)	
Income, quartiles, *N* (%)					<0.001
1st (highest)	4,094 (24.9)	973 (27.8)	729 (25.2)	5,071 (29.4)	
2nd	3,450 (21.0)	671 (19.2)	564 (19.5)	3,415 (19.8)	
3rd	3,207 (19.5)	702 (20.1)	576 (19.9)	3,207 (18.6)	
4th (lowest)	5,711 (34.7)	1,151 (32.9)	1,021 (35.3)	5,553 (32.2)	
Smoking status, *N* (%)					<0.001
Never-smoker	15,950 (96.9)	3,422 (97.9)	2,777 (96.1)	16,675 (96.7)	
Past smoker	318 (1.9)	44 (1.3)	80 (2.8)	386 (2.2)	
Current smoker	194 (1.2)	31 (0.9)	33 (1.1)	185 (1.1)	
Alcohol consumption [times per week], *N* (%)					<0.001
0	14,978 (91.0)	3,247 (92.8)	2,523 (87.2)	15,318 (88.8)	
1–2	1,288 (7.8)	212 (6.1)	319 (11.0)	1,737 (10.1)	
3–4	134 (0.8)	28 (0.8)	37 (1.3)	128 (0.7)	
≥5	62 (0.4)	10 (0.3)	11 (0.4)	63 (0.4)	
Physical activity [times per week], *N* (%)					<0.001
0	7,536 (45.8)	1,381 (39.5)	1,197 (41.4)	5,955 (34.5)	
1–2	2,245 (13.6)	458 (13.1)	419 (14.5)	2,794 (16.2)	
3–4	2,404 (14.6)	545 (15.6)	491 (17.0)	3,131 (18.2)	
≥5	4,277 (26.0)	1,113 (31.8)	783 (27.1)	5,366 (31.1)	
BMI [kg/m^2^], mean (SD)	26.43 (2.55)	22.44 (0.85)	24.16 (1.01)	20.92 (1.50)	<0.001
BMI [kg/m^2^], *N* (%)					<0.001
<23.0	186 (1.1)	2,467 (70.6)	209 (7.2)	16,083 (93.3)	
≥23.0	16,276 (98.9)	1,030 (29.5)	2,681 (92.8)	1,163 (6.7)	
Charlson comorbidity index, *N* (%)					<0.001
≤2	6,069 (36.9)	1,462 (41.8)	1,377 (47.6)	8,926 (51.8)	
3–4	6,888 (41.8)	1,438 (41.1)	1,100 (38.1)	6,386 (37.0)	
≥5	3,505 (21.3)	597 (17.1)	413 (14.3)	1,934 (11.2)	
Chemotherapy^a^, *N* (%)	9,907 (60.2)	2,225 (63.6)	1,650 (57.1)	9,702 (56.3)	<0.001
Doxorubicin	4,608 (28.0)	1,018 (29.1)	743 (25.7)	5,083 (29.5)	<0.001
Cyclophosphamide	9,034 (54.9)	2,036 (58.2)	1,519 (52.6)	9,020 (52.3)	<0.001
Paclitaxel	1,661 (10.1)	363 (10.4)	275 (9.5)	1,454 (8.4)	<0.001
Radiation therapy, *N* (%)	11,197 (68.0)	2,448 (70.0)	1,940 (67.1)	11,550 (67.0)	0.003
Hormone therapy^b^, *N* (%)	12,163 (73.9)	2,563 (73.3)	2,191 (75.8)	12,820 (74.3)	0.093
Tamoxifen	6,878 (41.8)	1,545 (44.2)	1,582 (54.7)	9,508 (55.1)	<0.001
Systolic BP [mmHg], mean (SD)	125.0 (14.7)	119.2 (14.9)	120.2 (13.6)	115.5 (14.0)	<0.001
Diastolic BP [mmHg], mean (SD)	77.0 (9.5)	73.7 (9.5)	74.9 (9.1)	72.1 (9.3)	<0.001
Total cholesterol [mg/dl], mean (SD)	194.0 (37.9)	191.7 (36.4)	196.5 (37.0)	191.5 (36.8)	<0.001
Fasting serum glucose [mg/dl], mean (SD)	102.5 (23.6)	97.8 (20.57)	96.8 (16.1)	93.7 (15.2)	<0.001

*p*-values calculated via Chi squared test for categorical variables and analysis of variance (ANOVA) for continuous variables.

SD, standard deviation; BMI, body mass index; BP, blood pressure.

^a^ cyclophosphamide, trastuzumab, doxorubicin, epirubicin, docetaxel, paclitaxel, and cisplatin.

^b^ tamoxifen, anastrozole, and letrozole.

Cardiovascular risks of the study population were observed, based on change (prior to and 5 years after diagnosis of breast cancer) in pLBMP, pASMP, and pBFMP (Table 2). Compared to those who continued to have low pLBMP and pASMP, those with persistently high pLBMP and pASMP had lower risks of CVD (aHR, 0.68 [95% CI, 0.53–0.87] and aHR, 0.60 [95% CI, 0.44–0.81], respectively). In contrast, both the High to Low and High to High pBFMP groups had higher risks of CVD (aHR, 1.44 [95% CI, 0.98–2.10] and aHR, 1.48 [95% CI, 1.15–1.89], respectively), compared to those who maintained a low pBFMP. Notably, those with increased (a low to high change) pBFMP had higher risk of CVD (aHR 1.51, [95% CI 0.99–2.31]. This pattern was also evident in Models 2, 3, and when High and Low groups were classified based on mean pLBMP, pASMP, and pBFMP of first and second health checkups (Suplementary Table S1).

**Table 2:** Hazard ratios for cardiovascular disease according to change in predicted body composition.

	Change in percentage of predicted body composition			
	Low % at baseline periods		High % at baseline periods	
	Low to Low	Low to High	High to Low	High to High
Change in pLBMP				
Study population, *N*	16,462	3,497	2,890	17,246
Percentage at baseline period [%], means (SD)	62.14 (1.87)	64.00 (0.84)	65.92 (0.81)	67.84 (2.01)
Percentage at follow-up period [%], means (SD)	62.19 (1.89)	66.05 (0.88)	64.01 (0.84)	68.00 (2.08)
CVD; events, *N* (%)	193 (1.17)	39 (1.12)	25 (0.87)	98 (0.57)
aHR (95% CI)				
Model 1	1.00 (reference)	1.01 (0.72, 1.43)	0.96 (0.63, 1.45)	0.68 (0.53, 0.87)**
	–	–	1.00 (reference)	0.71 (0.46, 1.10)
Model 2	1.00 (reference)	1.04 (0.73, 1.46)	0.97 (0.64, 1.47)	0.69 (0.54, 0.89)**
	–	–	1.00 (reference)	0.72 (0.46, 1.10)
Model 3	1.00 (reference)	1.07 (0.76, 1.52)	0.97 (0.64, 1.48)	0.72 (0.56, 0.94)*
	–	–	1.00 (reference)	0.74 (0.48, 1.16)
Change in pASMP				
Study population, *N*	20,505	2,597	3,304	13,689
Percentage at baseline period [%], means (SD)	24.77 (0.80)	25.62 (0.31)	26.39 (0.33)	27.12 (0.77)
Percentage at follow-up period [%], means (SD)	24.71 (0.82)	26.38 (0.32)	25.58 (0.34)	27.08 (0.77)
CVD; events, *N* (%)	257 (1.25)	20 (0.77)	16 (0.48)	62 (0.45)
aHR (95% CI)				
Model 1	1.00 (reference)	0.78 (0.49, 1.23)	0.55 (0.33, 0.92)*	0.60 (0.44, 0.81)***
	–	–	1.00 (reference)	1.09 (0.63, 1.89)
Model 2	1.00 (reference)	0.78 (0.50, 1.24)	0.56 (0.34, 0.93)*	0.61 (0.46, 0.83)**
	–	–	1.00 (reference)	1.10 (0.63, 1.91)
Model 3	1.00 (reference)	0.81 (0.51, 1.28)	0.56 (0.34, 0.94)*	0.65 (0.48, 0.87)**
	–	–	1.00 (reference)	1.15 (0.66, 1.99)
Change in pBFMP				
Study population, *N*	17,930	2,842	3,439	15,884
Percentage at baseline period [%], means (SD)	31.11 (2.01)	33.05 (0.82)	34.97 (0.80)	36.74 (1.82)
Percentage at follow-up period [%], means (SD)	30.96 (2.07)	34.95 (0.80)	32.92 (0.89)	36.70 (1.84)
CVD; events, *N* (%)	103 (0.57)	27 (0.95)	37 (1.08)	188 (1.18)
aHR (95% CI)				
Model 1	1.00 (reference)	1.51 (0.99, 2.31)	1.44 (0.98, 2.10)	1.48 (1.15, 1.89)**
	–	–	1.00 (reference)	1.03 (0.72, 1.46)
Model 2	1.00 (reference)	1.50 (0.98, 2.30)	1.43 (0.98, 2.08)	1.44 (1.12, 1.85)**
	–	–	1.00 (reference)	1.01 (0.71, 1.44)
Model 3	1.00 (reference)	1.44 (0.94, 2.21)	1.42 (0.97, 2.07)	1.38 (1.07, 1.78)*
	–	–	1.00 (reference)	0.97 (0.68, 1.39)

Adjusted hazard ratios calculated by multivariable Cox proportional hazards regression analysis after adjustments for the following covariates.

Model 1: age, income, chemotherapy, radiation therapy, hormone therapy, and Charlson comorbidity index.

Model 2: Model 1 + smoking status, alcohol consumption, and physical activity.

Model 3: Model 2 + blood pressures, total cholesterol, and fasting serum glucose.

Acronyms: cardiovascular disease (CVD); predicted lean body mass percentage (pLBMP); predicted appendicular skeletal mass percentage (pASMP); predicted body fat mass percentage (pBFMP); standard deviation (SD); hazard ratio (HR); confidence interval (CI).

* *p*-value < 0.05, **p-value < 0.01, and ***p-value < 0.001.

Results from the stratified analysis on the association of change in predicted body composition with CVD according to subgroups of age, CCI, and treatment pattern are shown in Table 3. In comparison to the Low to Low group, subjects in the High to High group, aged 60 or above, with less comorbidities, and who had received hormone therapy, had significantly lower (for pLBMP and pASMP) or higher risk (for pBFMP) of CVD. These risks were not affected by subgroups of age, CCI, or treatment pattern (*p* for interaction >0.05).

**Table 3:** Stratified analysis of hazard ratios for cardiovascular disease according to the subgroups of covariates.

	Percentage of predicted body composition				*p_interaction_*
	Low % at baseline periods		High % at baseline periods		
	Low to Low	Low to High	High to Low	High to High	
pLBMP					
Age [years]					0.391
40–49	1.00 (reference)	–	0.70 (0.08, 5.97)	0.56 (0.18, 1.76)	
50–59	1.00 (reference)	0.96 (0.50, 1.85)	1.08 (0.56, 2.08)	0.76 (0.51, 1.14)	
≥60	1.00 (reference)	1.05 (0.69, 1.60)	0.88 (0.50, 1.55)	0.64 (0.436, 0.90)*	
Charlson comorbidity index					0.990
1–3	1.00 (reference)	0.99 (0.62, 1.58)	0.97 (0.57, 1.64)	0.71 (0.52, 0.97)*	
≥4	1.00 (reference)	1.10 (0.66, 1.84)	0.98 (0.49, 1.96)	0.70 (0.46, 1.06)	
Treatment pattern					
Radiation therapy					0.836
Yes	1.00 (reference)	1.03 (0.66, 1.61)	0.95 (0.54, 1.66)	0.74 (0.54, 1.03)	
No	1.00 (reference)	1.09 (0.64, 1.87)	1.00 (0.53, 1.88)	0.63 (0.42, 0.93)*	
Chemotherapy					0.789
Yes	1.00 (reference)	1.09 (0.68, 1.73)	1.23 (0.71, 2.12)	0.75 (0.53, 1.07)	
No	1.00 (reference)	0.98 (0.58, 1.64)	0.71 (0.37, 1.38)	0.63 (0.44, 0.91)**	
Hormone therapy					0.520
Yes	1.00 (reference)	1.14 (0.76, 1.72)	1.04 (0.63, 1.71)	0.64 (0.47, 0.89)**	
No	1.00 (reference)	0.85 (0.45, 1.62)	0.80 (0.37, 1.76)	0.78 (0.52, 1.17)	
pASMP					
Age [years]					0.069
40–49	1.00 (reference)	0.67 (0.08, 5.43)	0.45 (0.06, 3.65)	0.37 (0.12, 1.16)	
50–59	1.00 (reference)	0.74 (0.36, 1.54)	0.42 (0.18, 0.98)*	0.68 (0.45, 1.02)	
≥60	1.00 (reference)	0.77 (0.42, 1.42)	0.66 (0.34, 1.28)	0.55 (0.35, 0.88)*	
Charlson comorbidity index					0.305
1–3	1.00 (reference)	0.77 (0.42, 1.39)	0.62 (0.34, 1.13)	0.61 (0.42, 0.88)**	
≥4	1.00 (reference)	0.85 (0.41, 1.74)	0.43 (0.16, 1.18)	0.67 (0.40, 1.11)	
Treatment pattern					
Radiation therapy					0.520
Yes	1.00 (reference)	0.57 (0.29, 1.12)	0.47 (0.23, 0.97)*	0.61 (0.42, 0.90)*	
No	1.00 (reference)	1.14 (0.61, 2.15)	0.69 (0.33, 1.42)	0.63 (0.39, 1.01)	
Chemotherapy					0.988
Yes	1.00 (reference)	0.76 (0.41, 1.41)	0.54 (0.25, 1.17)	0.69 (0.46, 1.03)	
No	1.00 (reference)	0.84 (0.43, 1.67)	0.58 (0.29, 1.15)	0.54 (0.35, 0.84)**	
Hormone therapy					0.894
Yes	1.00 (reference)	0.66 (0.36, 1.23)	0.64 (0.35, 1.16)	0.60 (0.41, 0.86)**	
No	1.00 (reference)	1.04 (0.52, 2.08)	0.40 (0.14, 1.08)	0.65 (0.39, 1.08)	
pBFMP					
Age [years]					0.350
40–49	1.00 (reference)	1.25 (0.15, 10.46)	3.52 (0.70, 17.76)	1.92 (0.61, 6.06)	
50–59	1.00 (reference)	1.77 (0.96, 3.28)	1.47 (0.78, 2.78)	1.33 (0.88, 2.01)	
≥60	1.00 (reference)	1.30 (0.70, 2.38)	1.38 (0.84, 2.25)	1.49 (1.08, 2.07)*	
Charlson comorbidity index					0.900
1–3	1.00 (reference)	1.45 (0.85, 2.46)	1.35 (0.82, 2.23)	1.42 (1.04, 1.94)*	
≥4	1.00 (reference)	1.53 (0.75, 3.11)	1.47 (0.82, 2.65)	1.41 (0.93, 2.12)	
Treatment pattern					
Radiation therapy					0.892
Yes	1.00 (reference)	1.38 (0.78, 2.42)	1.27 (0.77, 2.09)	1.34 (0.98, 1.86)	
No	1.00 (reference)	1.68 (0.88, 3.20)	1.73 (0.97, 3.09)	1.57 (1.06, 2.31)*	
Chemotherapy					0.914
Yes	1.00 (reference)	1.78 (1.01, 3.12)*	1.39 (0.84, 2.32)	1.36 (0.96, 1.92)	
No	1.00 (reference)	1.20 (0.63, 2.31)	1.46 (0.83, 2.58)	1.54 (1.08, 2.20)*	
Hormone therapy					0.422
Yes	1.00 (reference)	1.60 (0.95, 2.71)	1.57 (0.98, 2.51)	1.55 (1.14, 2.13)**	
No	1.00 (reference)	1.31 (0.64, 2.71)	1.22 (0.64, 2.34)	1.26 (0.84, 1.90)	

Adjusted hazard ratios calculated by multivariable Cox proportional hazards regression analysis after adjustments for the following covariates: age, income, chemotherapy, radiation therapy, hormone therapy, Charlson comorbidity index, smoking status, alcohol consumption, and physical activity.

Acronyms: cardiovascular disease (CVD); predicted lean body mass percentage (pLBMP); predicted appendicular skeletal mass percentage (pASMP); predicted body fat mass percentage (pBFMP); hazard ratio (HR); confidence interval (CI).

* *p*-value < 0.05, **p-value < 0.01, and ***p-value < 0.001 compared to the Low to Low group.

**Figure 2:**
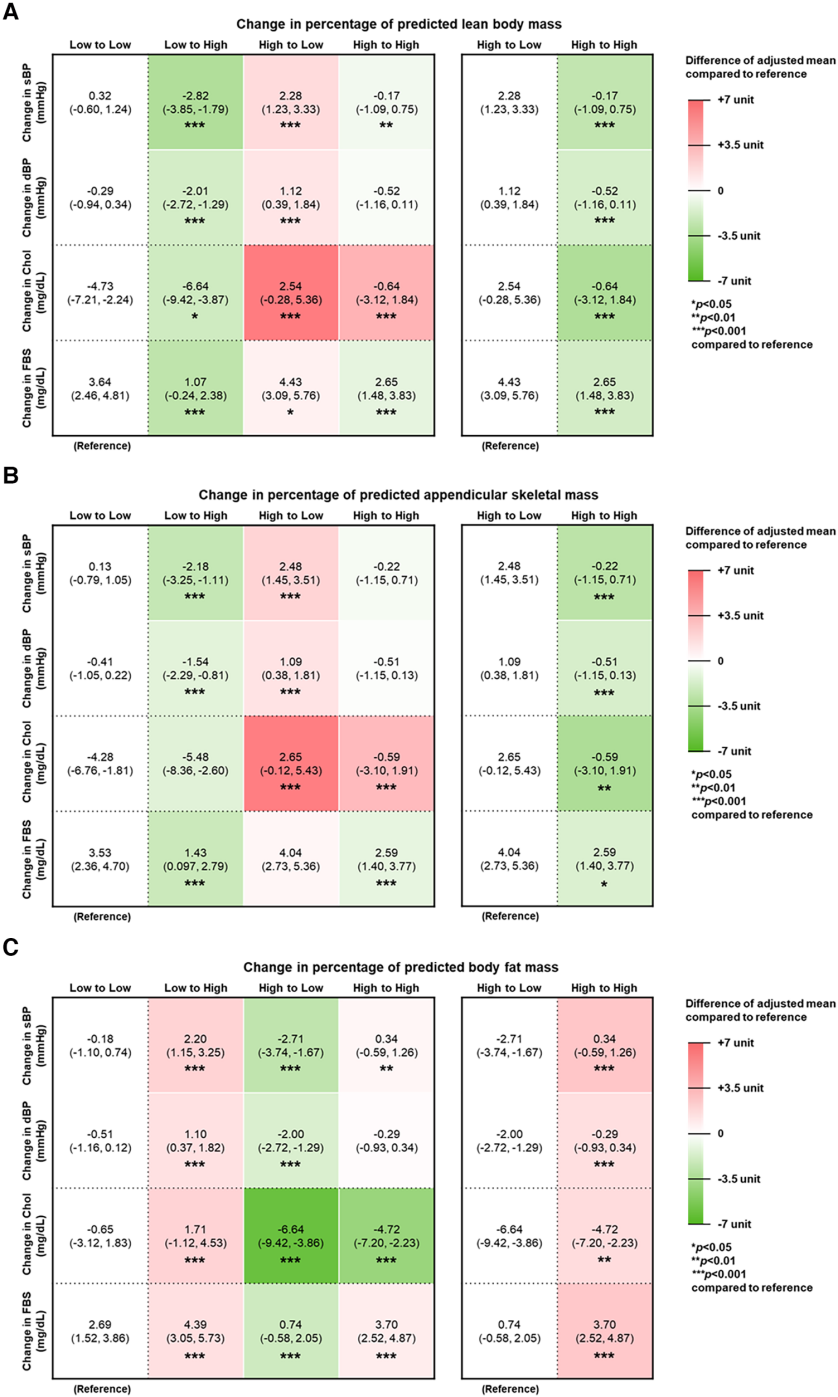
Changes in metabolic factors related to CVD according to predicted body composition. Adjusted mean of change in systolic blood pressure (sBP), diastolic blood pressure (dBP), total cholesterol (TC), and fasting serum glucose (FSG) according to changes in percentage of predicted (**A**) lean body mass, (**B**) appendicular skeletal muscle mass, and (**C**) body fat mass. Adjusted mean and 95% confidence interval (95% CI) calculated by multiple linear regression after adjustments for the following covariates: age, income, chemotherapy, radiation therapy, hormone therapy, Charlson comorbidity index, smoking status, alcohol consumption, and physical activity. The values inside the table indicate adjusted means and 95% CI. The relative difference of adjusted mean compared to the reference group is expressed in respective heat maps. Red indicates higher value, whereas green indicates lower value.

Figure 2 depicts the increased or decreased levels (changes) in metabolic factors, namely, that of blood pressure, fasting serum glucose, and total cholesterol, in relation to predicted body composition. Pertaining to changes in pLBMP and pASMP, the Low to High group showed decreased sBP, dBP, total cholesterol, and FSG, compared to the Low to Low group. Pertaining to change in pBFMP, the Low to High group showed increased sBP, dBP, total cholesterol, and FSG (aMean, 2.20 [95% CI, 1.15∼3.25]; aMean, 1.10 [95% CI, 0.37–1.82]; aMean, 1.71 [95% CI, −1.12∼4.53]; and aMean, 4.39 [95% CI, 3.05–5.73]. This pattern was also evident when comparing within those who initially had high pLBMP, pASMP, or pBFMP. Those who maintained high pLBMP and pASMP had decreased metabolic markers compared to those who changed from high to low pLBMP and pASMP; those who maintained high pBFMP had increased metabolic markers compared to those who changed from high to low pBFMP. In the case of change in total cholesterol, total cholesterol decreased less in the High to High group of pLBMP and pASMP (aMean, −0.64 [95% CI, −3.12∼1.84] and aMean, −0.59 [95% CI, −3.10∼1.91], respectively), compared to that of the Low to Low group while total cholesterol decreased more in the High to High group of pBFMP (aMean, −4.72 [95% CI, −7.20∼−2.23), compared to that of the Low to Low group.

## Discussion

In summary, having a persistently high predicted LBMP or ASMP among breast cancer survivors was associated with lower CVD risk than a persistently low predicted LBMP or ASMP, respectively. Conversely, persistently high predicted BFMP among breast cancer survivors was associated with higher CVD risk than persistently low predicted BFMP. Additionally, those with a low to high change in pBFMP had a higher risk of CVD than those with persistently low pBFMP. Changes in body composition were accompanied by changes in metabolic markers. To the best of the authors' knowledge, this is the first study to demonstrate the CVD risk with changes in body composition, by distinguishing muscle mass and fat mass as predicted LBMP, ASMP, and BFMP in breast cancer survivors.

Our findings are consistent with a previous study that examined adiposity from CT scans taken near diagnosis and subsequent CVD risk in breast cancer survivors (13). In this previous study, visceral and intramuscular adiposity were associated with increased CVD incidence, though only adiposity was examined and at one-time point. Similarly, central obesity was associated with higher all-cause and breast cancer–specific mortality among Black breast cancer survivors (31); CVD nor CVD-related mortality was observed in this study. In a cross-sectional study of anthracycline chemotherapy for breast cancer patients, greater thigh muscle fatty infiltration was associated with impaired oxygen extraction, which is a predictor of CVD morbidity and mortality (32). According to a study that observed for change in BMI and waist circumference between diagnosis and 24 months post-diagnosis in early-stage breast cancer patients, weight change was not associated with risk of CVD, while any elevation in waist circumference was associated with increased risk of CVD (12). As noted by the author, BMI and weight change do not accurately represent body composition, underscoring the significance of examining body composition and changes in body composition.

Although a low to high change in pBFMP showed a higher risk of CVD than those with persistently low pBFMP, so did that of a high to low change in pBFMP (both were not significant). Having a high body fat mass percentage before initial breast cancer diagnosis or at any timepoint in cancer survivorship may be decisive. Therefore, current results should be interpreted with discretion, and further examination should be carried on CVD risk according to changes in body fat mass percentage respective to muscle mass percentage as well as changes in metabolic markers.

The associations of changes in predicted LBMP, ASMP, and BFMP with CVD risk were statistically significant in the older or healthier individuals, which is consistent with findings from a previous study on young adults (14). Furthermore, these changes were significant in breast cancer survivors who had a history of hormone therapy. Although cardiovascular effects vary based on the type and combination of hormone therapy, hormone therapy is largely associated with an increased risk of stroke, coronary heart disease, and notably, venous thromboembolism (33, 34). Therefore, those who received hormone therapy, may benefit, in terms of CVD prevention, from changes in LBMP, ASMP, and BFMP.

The metabolic changes associated with changes in body composition have been studied before (25). Similar findings in breast cancer survivors were observed in our study. Increased or persistently high (compared to a high to low change in) predicted LBMP and ASMP were associated with decreased blood pressure, fasting serum glucose, and total cholesterol. Conversely, increased or persistently high (compared to a high to low change in) predicted BFMP was associated with increased blood pressure, fasting serum glucose, and total cholesterol. Indeed, adipose tissue inflammation includes insulin resistance, alterations in lipid metabolism, and blood pressure regulation, favoring endothelial dysfunction and atherogenesis (15). Skeletal muscle tissue, another endocrine organ that produces myokines, is also involved in the complex network related to metabolic functions (35). Myokines such as irisin and fibroblast growth factor-21 (FGF-21) are induced by physical exercise and increase insulin sensitivity and in the case of FGF-21, acts on lipolysis; apelin not only has an anti-inflammatory role but also controls cardiac muscles and blood pressure. However, further research is needed to investigate the joint effects of adiposity, muscle mass, and the crosstalk between respective cytokines (36).

Our study is not without limitations. Prediction equations were used to measure body composition, which may be imperfect for patients with cancer and difficult to apply to other ethnicities. However, a previous large validation study in the same ethnic group was conducted and showed high predictive values. In addition, in previous studies using the same equations, similar changes in body composition (increase in body fat mass and decrease in appendicular skeletal mass or lean body mass) were associated with an increased risk of metabolic syndrome and CVD in young adults (14, 25). Utilizing prediction questions may not only be less costly but also overcome the limitations of applying independent anthropometric indices (cut-offs). Furthermore, the study was not inclusive of cardiovascular biomarkers associated with adiposity (37). Therefore, future exploration on delineating associated immunometabolic pathways may provide insight on the progression from changes in body composition to CVD in breast cancer survivors. A major strength of our study is the large study population, considering that it was limited to 5-year breast cancer survivors. Changes in predicted body composition were observed as percentages, which may be clinically more meaningful, and were inclusive of both adiposity and muscle mass. Though it is unknown whether positive changes in body composition may reverse CVD risk, body composition is not unmodifiable, with lifestyle modification including physical activity and adequate nutrition; adhering to a healthy lifestyle showed to be particularly beneficial for those with a high genetic risk for CVD (38). In conclusion, among breast cancer survivors, persistently high muscle mass, represented as predicted LBMP or ASMP, was associated with lower CVD risk. Preventing an increase in fat mass may be beneficial in preventing CVD in breast cancer survivors, as a part of cancer survivorship.

## Data Availability

The data analyzed in this study is subject to the following licenses/restrictions: The datasets generated and/or analyzed during the current study are not publicly available due to institutional policy but are available from the corresponding author upon reasonable request. Requests to access these datasets should be directed to Jihun Song, jih2616@naver.com.
