# Supplementary material for: Changes in body composition and subsequent cardiovascular disease risk among 5-year breast cancer survivors
Source: Front Cardiovasc Med. 2023 Nov 20;10:1259292. doi: 10.3389/fcvm.2023.1259292 (PMC10694451; doi:10.3389/fcvm.2023.1259292)
Supplement: Supplementary file 1 [file Table1.docx]

**Table S1.** Sensitive analysis: Hazard ratios for cardiovascular disease according to change in predicted body composition.

|  | **Change in percentage of predicted body composition** | | | |
| --- | --- | --- | --- | --- |
|  | **Low % at baseline periods** | | **High % at baseline periods** | |
|  | **Low to Low** | **Low to High** | **High to Low** | **High to High** |
| **Change in pLBMP** |  |  |  |  |
| Study population, N | 16,871 | 3,176 | 3,177 | 16,871 |
| Percentage at baseline period [%], means (SD) | 62.18 (1.87) | 64.04 (0.83) | 65.95 (0.83) | 67.88 (2.01) |
| Percentage at follow-up period [%], means (SD) | 62.26 (1.92) | 66.17 (0.86) | 64.12 (0.86) | 68.06 (2.05) |
| CVD; events, N (%) | 201 (1.19) | 31 (0.98) | 29 (0.91) | 94 (0.56) |
| aHR (95% CI) | 1.00 (reference) | 0.88 (0.60, 1.29) | 1.00 (0.68, 1.49) | 0.66 (0.52, 0.86)** |
|  | - | - | 1.00 (reference) | 0.66 (0.44, 1.00) |
| **Change in pASMP** |  |  |  |  |
| Study population, N | 17,082 | 2,966 | 2,966 | 17,081 |
| Percentage at baseline period [%], means (SD) | 24.59 (0.75) | 25.37 (0.31) | 26.13 (0.32) | 26.93 (0.81) |
| Percentage at follow-up period [%], means (SD) | 24.52 (0.76) | 26.08 (0.33) | 25.29 (0.31) | 26.87 (0.82) |
| CVD; events, N (%) | 232 (1.36) | 19 (0.64) | 16 (0.54) | 88 (0.52) |
| aHR (95% CI) | 1.00 (reference) | 0.60 (0.37, 0.95)* | 0.56 (0.34, 0.94)* | 0.64 (0.49, 0.84)** |
|  | - | - | 1.00 (reference) | 1.15 (0.67, 1.96) |
| **Change in pBFMP** |  |  |  |  |
| Study population, N | 16,916 | 3,132 | 3,132 | 16,915 |
| Percentage at baseline period [%], means (SD) | 30.98 (1.99) | 32.89 (0.83) | 34.82 (0.79) | 36.62 (1.84) |
| Percentage at follow-up period [%], means (SD) | 30.81 (2.003) | 34.73 (0.83) | 32.69 (0.86) | 36.55 (1.89) |
| CVD; events, N (%) | 93 (0.55) | 26 (0.83) | 34 (1.09) | 202 (1.19) |
| aHR (95% CI) | 1.00 (reference) | 1.38 (0.89, 2.13) | 1.49 (1.00, 2.21)* | 1.52 (1.18, 1.96)** |
|  | - | - | 1.00 (reference) | 1.02 (0.71, 1.47) |

Adjusted hazard ratios calculated by multivariable Cox proportional hazards regression analysis after adjustments for the following covariates.: age, income, chemotherapy, radiation therapy, hormone therapy, Charlson comorbidity index, smoking status, alcohol consumption, and physical activity.

Acronyms: cardiovascular disease (CVD); predicted lean body mass percentage (pLBMP); predicted appendicular skeletal mass percentage (pASMP); predicted body fat mass percentage (pBFMP); standard deviation (SD); hazard ratio (HR); confidence interval (CI).

**p*-value<0.05, ***p*-value<0.01, and ****p*-value<0.001

**Table S2.** Changes in metabolic risk factors according to predicted body composition.

|  | **Change in percentage of predicted body composition** | | | |
| --- | --- | --- | --- | --- |
|  | **Low % at baseline periods** | | **High % at baseline periods** | |
|  | **Low to Low** | **Low to High** | **High to Low** | **High to High** |
| **Change in pLBMP** |  |  |  |  |
| **Study population^a^** | 16,458 | 3,497 | 2,887 | 17,243 |
| **Change in Systolic BP [mmHg]** |  |  |  |  |
| at baseline period, means (SD) | 124.9 (15.2) | 122.2 (15.1) | 118.1 (14.1) | 115.8 (13.9) |
| at follow-up period, means (SD) | 125.0 (14.7) | 119.2 (14.9) | 120.2 (13.6) | 115.5 (14.0) |
| Change in systolic BP, means (SD) | 0.1 (16.4) | -3.1 (15.7) | 2.2 (14.8) | -0.3 (14.1) |
| aMean (95% CI) | 0.32 (-0.60, 1.24) | -2.82 (-3.85, -1.79) | 2.28 (1.23, 3.33) | -0.17 (-1.09, 0.75) |
| *p*-value | Reference | <0.001 | <0.001 | 0.004 |
|  | - | - | Reference | <0.001 |
| **Change in Diastolic BP [mmHg]** |  |  |  |  |
| at baseline period, means (SD) | 77.1 (10.0) | 75.5 (9.8) | 73.4 (9.7) | 72.1 (9.4) |
| at follow-up period, means (SD) | 77.0 (9.5) | 73.7 (9.5) | 74.9 (9.1) | 72.1 (9.3) |
| Change in systolic BP, means (SD) | -0.1 (11.1) | -1.8 (10.4) | 1.5 (10.7) | -0.1 (10.0) |
| aMean (95% CI) | -0.29 (-0.94, 0.34) | -2.01 (-2.72, -1.29) | 1.12 (0.39, 1.84) | -0.52 (-1.16, 0.11) |
| *p*-value | Reference | <0.001 | <0.001 | 0.061 |
|  | - | - | Reference | <0.001 |
| **Change in Total cholesterol [mg/dL]** |  |  |  |  |
| at baseline period, means (SD) | 203.3 (41.0) | 202.3 (36.8) | 196.7 (36.5) | 194.3 (34.5) |
| at follow-up period, means (SD) | 194.0 (37.9) | 191.7 (36.4) | 196.5 (37.0) | 191.5 (36.8) |
| Change in Total cholesterol, means (SD) | -9.3 (45.6) | -10.6 (40.5) | -0.3 (40.0) | -2.8 (37.6) |
| aMean (95% CI) | -4.73 (-7.21, -2.24) | -6.64 (-9.42, -3.87) | 2.54 (-0.28, 5.36) | -0.64 (-3.12, 1.84) |
| *p*-value | Reference | 0.012 | <0.001 | <0.001 |
|  | - | - | Reference | <0.001 |
| **Change in Fasting serum glucose [mg/dL]** |  |  |  |  |
| at baseline period, means (SD) | 100.1 (23.4) | 98.2 (20.9) | 93.7 (17.1) | 92.4 (14.8) |
| at follow-up period, means (SD) | 102.5 (23.6) | 97.8 (20.7) | 96.8 (16.1) | 93.7 (15.2) |
| Change in Fasting serum glucose, means (SD) | 2.3 (23.4) | -0.6 (19.1) | 3.1 (15.9) | 1.3 (15.3) |
| aMean (95% CI) | 3.64 (2.46, 4.81) | 1.07 (-0.24, 2.38) | 4.43 (3.09, 5.76) | 2.65 (1.48, 3.83) |
| *p*-value | Reference | <0.001 | 0.046 | <0.001 |
|  | - | - | Reference | <0.001 |
| **Change in pASMP** |  |  |  |  |
| **Study population^a^** | 20,499 | 2,597 | 3,302 | 13,687 |
| **Change in Systolic BP [mmHg]** |  |  |  |  |
| at baseline period, means (SD) | 124.5 (15.3) | 120.0 (14.4) | 117.0 (13.7) | 114.8 (13.5) |
| at follow-up period, means (SD) | 124.3 (14.8) | 117.5 (14.5) | 119.3 (13.7) | 114.4 (13.5) |
| Change in systolic BP, means (SD) | -0.1 (16.3) | -2.5 (14.9) | 2.3 (14.2) | -0.4 (14.0) |
| aMean (95% CI) | 0.13 (-0.79, 1.05) | -2.18 (-3.25, -1.11) | 2.48 (1.45, 3.51) | -0.22 (-1.15, 0.71) |
| *p*-value | Reference | <0.001 | <0.001 | 0.057 |
|  | - | - | Reference | <0.001 |
| **Change in Diastolic BP [mmHg]** |  |  |  |  |
| at baseline period, means (SD) | 76.7 (10.0) | 74.6 (9.8) | 72.9 (9.5) | 71.7 (9.3) |
| at follow-up period, means (SD) | 76.4 (9.5) | 73.3 (9.6) | 74.5 (9.4) | 71.7 (9.3) |
| Change in systolic BP, means (SD) | -0.3 (11.0) | -1.3 (10.4) | 1.5 (10.4) | 0.0 (9.9) |
| aMean (95% CI) | -0.41 (-1.05, 0.22) | -1.54 (-2.29, -0.81) | 1.09 (0.38, 1.81) | -0.51 (-1.15, 0.13) |
| *p*-value | Reference | <0.001 | <0.001 | 0.438 |
|  | - | - | Reference | <0.001 |
| **Change in Total cholesterol [mg/dL]** |  |  |  |  |
| at baseline period, means (SD) | 203.7 (40.4) | 200.6 (36.1) | 196.1 (35.0) | 191.9 (33.4) |
| at follow-up period, means (SD) | 194.5 (37.9) | 192.1 (35.9) | 196.3 (37.4) | 189.7 (36.3) |
| Change in Total cholesterol, means (SD) | -9.2 (45.1) | -8.5 (39.2) | 0.2 (38.0) | -2.2 (36.8) |
| aMean (95% CI) | -4.28 (-6.76, -1.81) | -5.48 (-8.36, -2.60) | 2.65 (-0.12, 5.43) | -0.59 (-3.10, 1.91) |
| *p*-value | Reference | 0.166 | <0.001 | <0.001 |
|  | - | - | Reference | <0.001 |
| **Change in Fasting serum glucose [mg/dL]** |  |  |  |  |
| at baseline period, means (SD) | 99.6 (22.9) | 96.3 (19.0) | 92.9 (15.7) | 91.8 (15.7) |
| at follow-up period, means (SD) | 101.7 (23.1) | 96.3 (18.6) | 95.5 (15.7) | 93.0 (14.1) |
| Change in Fasting serum glucose, means (SD) | 2.1 (22.7) | -0.0 (17.5) | 2.7 (16.7) | 1.2 (14.5) |
| aMean (95% CI) | 3.53 (2.36, 4.70) | 1.43 (0.097, 2.79) | 4.04 (2.73, 5.36) | 2.59 (1.40, 3.77) |
| *p*-value | Reference | <0.001 | 0.160 | <0.001 |
|  | - | - | Reference | 0.023 |
| **Change in pBFMP** |  |  |  |  |
| **Study population^a^** | 17,927 | 2,839 | 3,438 | 15,881 |
| **Change in Systolic BP [mmHg]** |  |  |  |  |
| at baseline period, means (SD) | 115.9 (13.9) | 118.3 (14.2) | 122.4 (15.0) | 125.0 (15.3) |
| at follow-up period, means (SD) | 115.6 (14.0) | 120.3 (13.6) | 119.4 (15.0) | 125.2 (14.7) |
| Change in systolic BP, means (SD) | -0.3 (14.2) | 2.1 (14.9) | -3.0 (15.6) | 0.1 (16.4) |
| aMean (95% CI) | -0.18 (-1.10, 0.74) | 2.20 (1.15, 3.25) | -2.71 (-3.74, -1.67) | 0.34 (-0.59, 1.26) |
| *p*-value | Reference | <0.001 | <0.001 | 0.003 |
|  | - | - | Reference | <0.001 |
| **Change in Diastolic BP [mmHg]** |  |  |  |  |
| at baseline period, means (SD) | 72.2 (9.4) | 73.5 (9.7) | 75.6 (9.8) | 77.2 (10.0) |
| at follow-up period, means (SD) | 72.2 (9.4) | 75.0 (9.2) | 73.8 (9.5) | 77.0 (9.5) |
| Change in systolic BP, means (SD) | -0.1 (10.0) | 1.5 (10.9) | -1.8 (10.3) | -0.1 (11.1) |
| aMean (95% CI) | -0.51 (-1.16, 0.12) | 1.10 (0.37, 1.82) | -2.00 (-2.72, -1.29) | -0.29 (-0.93, 0.34) |
| *p*-value | Reference | <0.001 | <0.001 | 0.060 |
|  | - | - | Reference | <0.001 |
| **Change in Total cholesterol [mg/dL]** |  |  |  |  |
| at baseline period, means (SD) | 194.5 (34.5) | 197.3 (36.9) | 202.3 (37.1) | 203.3 (41.0) |
| at follow-up period, means (SD) | 191.7 (36.8) | 196.3 (37.1) | 191.7 (36.2) | 193.9 (38.0) |
| Change in Total cholesterol, means (SD) | -2.8 (37.7) | -1.1 (40.3) | -10.6 (40.6) | -9.4 (45.7) |
| aMean (95% CI) | -0.65 (-3.12, 1.83) | 1.71 (-1.12, 4.53) | -6.64 (-9.42, -3.86) | -4.72 (-7.20, -2.23) |
| *p*-value | Reference | <0.001 | <0.001 | <0.001 |
|  | - | - | Reference | 0.013 |
| **Change in Fasting serum glucose [mg/dL]** |  |  |  |  |
| at baseline period, means (SD) | 92.5 (14.8) | 93.7 (17.5) | 98.4 (21.3) | 100.3 (23.5) |
| at follow-up period, means (SD) | 93.9 (15.4) | 96.8 (16.4) | 97.8 (20.1) | 102.7 (23.7) |
| Change in Fasting serum glucose, means (SD) | 1.3 (15.4) | 3.1 (1.63) | -0.7 (19.6) | 2.4 (23.5) |
| aMean (95% CI) | 2.69 (1.52, 3.86) | 4.39 (3.05, 5.73) | 0.74 (-0.58, 2.05) | 3.70 (2.52, 4.87) |
| *p*-value | Reference | <0.001 | <0.001 | <0.001 |
|  | - | - | Reference | <0.001 |

Adjusted mean calculated by multiple linear regression analysis after adjustments for the following covariates: age, income, chemotherapy, radiation therapy, hormone therapy, Charlson comorbidity index, smoking status, alcohol consumption, and physical activity.

^a^N=40,085 (a total of 10 cancer survivors excluded due to missing value at health screening, prior to the date of cancer diagnosis)

Acronyms: standard deviation (SD); blood pressure (BP); adjusted mean (aMean); confidence interval (CI).
